# The ripple effect of stigmatization on children with congenital hypospadias: impacts on psychological and behavioral aspects

**DOI:** 10.3389/fpubh.2026.1743923

**Published:** 2026-02-18

**Authors:** Wei Zhang, Lin Mo, Tao Liu

**Affiliations:** 1 Department of Operating, Children’s Hospital of Chongqing Medical University, National Clinical Research Center for Children and Adolescents’ Health and Diseases, Ministry of Education Key Laboratory of Child Development and Disorders, Chongqing Key Laboratory of Child Neurodevelopment and Cognitive Disorders, Chongqing, China; 2 Department of Outpatient, Children’s Hospital of Chongqing Medical University, National Clinical Research Center for Children and Adolescents’ Health and Diseases, Ministry of Education Key Laboratory of Child Development and Disorders, Chongqing Key Laboratory of Child Neurodevelopment and Cognitive Disorders, Chongqing, China

**Keywords:** behavioral problems, children, hypospadias, influencing factors, nursing

## Abstract

**Objective:**

To investigate the status of behavioral problems in children with congenital hypospadias and analyze their influencing factors, providing a scientific basis for improving behavioral issues in these children.

**Methods:**

From May to October 2024, 143 children with hypospadias and their families were recruited using convenience sampling. Assessments were conducted using a general information questionnaire and the Conners Parent Rating Scale. Univariate and multivariate logistic analyses were employed to identify the influencing factors of behavioral problems in children with congenital hypospadias.

**Results:**

The incidence rate of behavioral problems among children with congenital hypospadias was 23.8%. The number of surgeries and urination status were identified as influencing factors for behavioral problems (*p* < 0.05).

**Conclusion:**

The incidence of behavioral problems is high among children with congenital hypospadias, and their levels are influenced by multiple factors. Medical professionals and family members should pay attention to the impact of the disease on the children’s psychological well-being. Interventions should be implemented based on the identified influencing factors to encourage positive and proactive treatment among the children and their families. This approach aims to reduce the impact of the disease on behavioral problems during the children’s growth process and enhance their social adaptability as adults.

## 1 Introduction

Congenital hypospadias is a condition characterized by abnormal development of the anterior urethra and urethral sponge, resulting in an atypical urethral opening position. Instead of opening at the normal tip of the penis, the urethra may open anywhere along the penis from the head to the perineum (1). The incidence rate of congenital hypospadias is approximately 0.32%, and it shows a rising trend year by year (2). Surgical intervention is the sole treatment for congenital hypospadias (3). However, the incidence of postoperative complications following hypospadias repair can be as high as 49.5% (4), with some cases requiring multiple surgeries for complete correction (5, 6). Surgery, as a negative stressor, can exert psychological adverse effects on pediatric patients, manifesting as behavioral issues such as anxiety, depression, and post-traumatic stress disorder (7). A literature review indicated that children who undergo hypospadias surgery are more prone to behavioral problems, including anxiety, depression, attention deficit, autism spectrum disorders, sexual psychopathology, and suicidal tendencies (8). Due to the unique characteristics of children’s psychological development, the manifestations of psychological and behavioral problems vary across different age groups. Infants and toddlers typically lack strong self-awareness and do not have a complete understanding of their condition. Preschool-aged children may exhibit marked social withdrawal, shyness, and timidity (9). School-aged children, on the other hand, may display behavioral issues such as anxiety, depression, heightened aggression, intense reactions, and attention deficits. These problems may even persist into adulthood, leading to inadequate social interaction skills, poor self-management abilities, and low social status (10). Currently, clinical research on children with hypospadias predominantly focuses on mitigating the short-term effects of postoperative pain (11, 12), while studies examining the psychological impact on behavior have mainly centered on the caregivers of affected children (13–16). Although previous studies by various scholars have confirmed the presence of diverse behavioral problems in children with hypospadias, the influencing factors remain unclear, hindering the development of targeted interventions for these behavioral issues. Therefore, this study aims to investigate the status of behavioral problems in children with congenital hypospadias and analyze the potential influencing factors associated with these problems. The objective is to provide initial evidence for subsequent research on children with congenital hypospadias and to assist in improving behavioral issues arising from the disease.

## 2 Materials and methods

### 2.1 Study type and design

This study is a cross-sectional investigation that randomly enrolled children aged 3–17 with hypospadias, conducting interviews and scale assessments with the children and their families to demonstrate the current status of behavioral issues in hypospadias patients and explore influencing factors.

### 2.2 Participants

From May to October 2024, a convenience sampling method was employed to select pediatric patients with congenital hypospadias and their family members from the Urology Department of a public tertiary-grade children’s hospital in Chongqing, China, as the survey subjects. Inclusion criteria: ① Diagnosed with congenital hypospadias; ② 3–17 years old; ③ The children and their families have normal communication abilities. Exclusion criteria: ① Have severe organic diseases; ② Have speech, hearing, intellectual, or cognitive functional impairments. Elimination criteria: The children or their families request to terminate the investigation. According to the sample size calculation formula (17):


n=max[(number of dimensions)×(10−15)]×[1+(15%−20%)]

The number of dimensions of the Conners scale is 6, and the calculated sample size should be 69–108 cases. In total, 143 children with hypospadias were investigated. All participants gave informed consent and voluntarily participated in this investigation. Among them, children under 8 years of age only require parental consent, while those over 8 years of age require both parental and child consent. This study was approved by the hospital ethics committee (initial review 2023), and the review number for research was (360).

### 2.3 Observation indicators

#### 2.3.1 Demographic and disease-related information

A self-designed general information questionnaire was utilized, encompassing both demographic data and disease-related information. This included details such as age, primary caregiver, disease classification, number of surgical procedures undergone, and urinary voiding conditions. Specifically, the urinary voiding conditions were categorized based on descriptions provided by family members in the admission records, ranked from most to least severe impact as follows: squatting urination, standing urination with dribbling, and standing urination without dribbling.

#### 2.3.2 Conners parent symptom questionnaire (PSQ)

The Conners Parent Symptom Questionnaire (PSQ) is employed to assess whether children aged 3 to 17 years exhibit psychological and behavioral problems, as well as to gauge the severity of such issues. The questionnaire comprises 48 multiple-choice questions, scored using a 4-point Likert scale (ranging from 0 to 3): 0 indicates the absence of the problem; 1 signifies occasional or mild manifestation; 2 denotes frequent or relatively severe occurrence; and 3 represents very common or highly severe presentation. The questions are categorized into six dimensions: conduct, learning, psychosomatic, impulsivity, anxiety, and hyperactivity. In 2001, Su et al. (18) in China developed the Chinese urban norm for the Conners Parent Symptom Questionnaire and Cronbach’s *α* is 0.925. This study will reference this norm, considering a score exceeding 1.5 points (or deviating by more than 2 standard deviations) on any dimension as indicative of abnormality.

### 2.4 Data collection

The questionnaire survey was conducted by a postgraduate student with 9 years of experience in pediatric nursing, under the guidance of a chief nurse who holds a National Level II Psychological Counselor qualification certificate. The primary survey method involved on-site interviews and distribution of paper PSQ questionnaires, with interviews taking place in the urology departments office. Researcher 1 was responsible for study introduction, answering questions, obtaining consent forms, and using standardized instructions and neutral explanatory language to guide participants in completing the questionnaires. The interview duration for each participant was approximately 30–55 min. Objective medical record data, such as the number of surgeries, disease classification, and urination status, were obtained from electronic medical records with the informed consent of the children’s parents. A total of 150 questionnaires were distributed. Among them, 7 questionnaires were excluded due to missing data or because the parents refused to complete them midway. Consequently, 143 valid questionnaires were included in the analysis, yielding an effective questionnaire recovery rate of 95.3%. Researcher 2 was responsible for entering all the participants information and questionnaire data, while Researcher 1 conducted a secondary verification of the data.

### 2.5 Statistical analysis

Data analysis was conducted using SPSS 21.0. Following normality testing, continuous variables not conforming to a normal distribution were reported as median (quartile), with between-group comparisons performed using the Mann–Whitney U test. Categorical variables were expressed as frequencies (*n*) or percentages (%), and chi-square tests were applied for group comparisons. Logistic regression analysis was utilized to identify factors influencing behavioral problems. Statistical significance was defined as *p* < 0.05.

## 3 Results

### 3.1 Demographic and disease-related characteristics

A total of 143 children with congenital hypospadias were included in this study. The general information is shown in Table 1.

**Table 1:** Demographic characteristics.

Variables	Number of cases	Positive (*n* = 34)	Negative (*n* = 109)	*χ* ^2^	*p*
Age, (*n* %)				1.014	0.828
3–5 years	48	10 (20.8)	38 (79.2)		
6–12 years	79	21 (26.6)	58 (73.4)		
13–15 years	13	3 (23.1)	10 (76.9)		
16–17 years	3	0 (0.0)	3 (100.0)		
Number of surgeries, (*n* %)				10.580	0.014
1 time	60	8 (13.3)	52 (86.7)		
2 times	41	9 (22.0)	32 (78.0)		
3 times	22	8 (36.4)	14 (63.6)		
>3 times	20	9 (45.0)	11 (55.0)		
Disease classification, (*n* %)				14.283	0.009
Balanic type	21	4 (19.0)	17 (81.0)		
Coronal type	31	2 (6.5)	29 (93.5)		
Penile type	59	17 (28.8)	42 (71.2)		
Penoscrotal type	21	6 (28.6)	15 (71.4)		
Scrotal type	5	4 (80.0)	1 (20.0)		
Perineal type	6	1 (16.7)	5 (83.3)		
Urination status, (*n* %)				10.218	0.006
Standing urination without dribbling	41	4 (9.8)	37 (90.2)		
Standing urination with dribbling	80	20 (25.0)	60 (75.0)		
Squatting urination	22	10 (45.5)	12 (54.5)		
Other surgical history, (*n* %)				0.857	0.355
Yes	29	5 (17.2)	24 (82.8)		
No	114	29 (25.4)	85 (74.6)		
Combining chronic diseases, (*n* %)				—	0.359
Yes	16	2 (12.5)	14 (87.5)		
No	127	32 (25.2)	95 (74.8)		
Days of indwelling catheter, (*n* %)				2.447	0.464
7 days	46	8 (17.4)	38 (82.6)		
8 days	82	21 (25.6)	61 (74.4)		
9 days	12	4 (33.3)	8 (66.7)		
≥10 days	3	1 (33.3)	2 (66.7)		
Residence, (*n* %)				0.858	0.354
Town	73	15 (20.5)	58 (79.5)		
Rural area	70	19 (27.1)	51 (72.9)		
Main caregivers, (*n* %)				1.701	0.398
Father	43	8 (18.6)	35 (81.4)		
Mother	95	24 (25.3)	71 (74.7)		
Others	5	2 (40.0)	3 (60.0)		

### 3.2 Incidence of behavioral problems in children with congenital hypospadias

Among the 143 children with hypospadias included in the study, 34 exhibited abnormal behavioral manifestations, yielding an incidence rate of 23.8%. A comparative analysis was conducted between the groups of children with negative and positive behavioral findings. No statistically significant differences were observed between the two groups in terms of age grouping, other surgical history, Combining chronic diseases, duration of indwelling urethral catheter, living environment, or main caregiver (*p* > 0.05). However, statistically significant differences were identified between the two groups regarding number of surgeries, disease classification and urination status (*p* < 0.05), as shown in Table 1.

The results revealed the following scores for various behavioral dimensions: conduct problems scored 9 (5.5, 12) points, learning problems scored 4 (2, 6) points, psychosomatic problems scored 0 (0, 1) point, impulsivity-hyperactivity scored 1 (0, 3) points, anxiety scored 2 (2, 4) points, and the hyperactivity index scored 7 (5, 10) points.

Among the 143 children with congenital hypospadias, 24 (16.8%) exhibited positive in only one behavioral dimension, 6 (4.2%) in two dimensions, 3 (2.1%) in three dimensions, and 1 (0.7%) in four dimensions. Specifically, 7 children (4.9%) tested positive for conduct problems, 9 (6.3%) for learning problems, 1 (0.7%) for psychosomatic problems, 1 (0.7%) for impulsivity-hyperactivity, 27 (18.9%) for anxiety, and 4 (2.8%) showed abnormal hyperactivity index scores.

### 3.3 Single factor analysis of influencing factors for behavioral problems

All observed indicators were included in a Univariate logistic regression analysis. The results indicated that the number of surgeries, disease classification, and urination status were significant factors influencing the positive rate of behavioral problems in the children (*p* < 0.05), as shown in Table 2.

**Table 2:** Single factor logistic regression of positive status in included children.

Variables	B	SE	Wald	*p*	*OR*	95% CI	
						Lower limit	Superior limit
Age	−0.009	0.287	0.001	0.976	0.991	0.565	1.740
Number of surgeries	0.576	0.184	9.753	0.002	1.779	1.239	2.554
Disease classification	0.334	0.164	4.161	0.041	1.397	1.013	1.925
Urination status	1.009	0.330	9.369	0.002	2.742	1.437	5.231
Other surgical history	−0.493	0.537	0.845	0.358	0.611	0.213	1.748
Combining chronic diseases	−0.858	0.783	1.200	0.273	0.424	0.091	1.968
Days of indwelling catheter	0.387	0.287	1.820	0.177	1.473	0.839	2.584
Domicile	0.365	0.395	0.853	0.356	1.441	0.664	3.125
Caregivers	0.454	0.394	1.326	0.250	1.574	0.727	3.407

### 3.4 Multivariate analysis of influencing factors for behavioral problems

Variables that demonstrated statistical significance in the univariate analysis were selected as independent variables. Based on the scores from the Conners Parent Rating Scale, behavioral problem status (negative = 1, positive = 0) was used as the dependent variable for binary logistic regression analysis. The assignment of independent variables is detailed as follows:

Number of surgeries (times): 1 time = 1; 2 times = 2; 3 times = 3; >3 times = 4.

Disease classification: Balanic type = 1; Coronal type = 2; Penile type = 3; Penoscrotal type = 4; Scrotal type = 5; Perineal type = 6.

Urination status: standing urination without dribbling = 0; standing urination with dribbling = 1; Squatting urination = 2.

The results revealed that the number of surgeries and urination status were significant influencing factors for positive behavioral problem status in children (*p* < 0.05). Specifically, both the number of surgeries and urination status were identified as risk factors for positive behavioral problem status (OR > 1, *p* < 0.05). As shown in the Table 3.

**Table 3:** Multivariate logistic regression of positive status in included children.

Metric	B	SE	Wald	*p*	*OR*	95% CI	
						Lower limit	Superior limit
Number of surgeries	0.440	0.196	5.025	0.025	1.552	1.057	2.280
Disease classification	0.070	0.185	0.144	0.705	1.073	0 0.747	1.540
Urination status	0.792	0.371	4.553	0.033	2.208	1.067	4.570
Constant	−3.901	0.844	21.350	0.000	0.020	/	/

## 4 Discussion

### 4.1 High incidence of behavioral problems in children with hypospadias

In this study, the incidence rate of positive behavioral problems among children with congenital hypospadias was 23.8%, aligning with the range (15%–65%) reported by Schönbucher et al. (19). As a congenital urological condition, our research found that behavioral issues in children with hypospadias become more pronounced after reaching school age. This is primarily attributed to the psychological development characteristics of children, who initially lack a distinct sense of gender identity. Upon entering school, they gradually recognize their differences from typically developing male peers, leading to the emergence of various emotional and behavioral problems. Specifically, 40% of school-aged children with hypospadias exhibit emotional symptoms such as anxiety, depression, low mood, and diminished interest (9). Behaviorally, they may demonstrate hyperactivity, aggression, heightened reactivity, and easy distractibility (20), with some displaying marked cross-gender behaviors (21). Compared to their peers, children with congenital hypospadias tend to encounter greater difficulties in behavior and social interaction after school age (22). Standing urination with dribbling and frequent incidents of wetting pants may prompt these children to intentionally withhold urination or avoid drinking water to reduce bathroom visits, fearing ridicule from peers. Such self-image challenges can hinder the development of their social competencies, including peer relationships and participation in social organizations (23, 24), and also low self-esteem (25). Additionally, multiple medical visits and treatments for hypospadias during childhood and adolescence can influence the development of coping strategies (emotional, behavioral, and cognitive responses to stressful situations), making them more prone to adopting avoidant coping mechanisms compared to healthy children (26). The behavioral manifestations observed across different age groups may stem from traditional, reserved Chinese parenting styles. Parents rarely initiate discussions on sexual and reproductive health, and children are often hesitant to communicate their disease-related distress. When children struggle to control urine flow and wet their pants, they may deliberately conceal their situation, while parents may choose to feign ignorance to protect their child’s self-esteem. On the other hand, some parents, feeling guilty due to the congenital nature of hypospadias, may be more inclined to tolerate and indulge their child’s negative emotions, potentially contributing to the high incidence of behavioral problems. Therefore, we recommend establishing support groups for families affected by hypospadias to foster mutual support and experience sharing, encouraging parents to prioritize their child’s psychological development. Additionally, hospitals could offer cognitive-behavioral therapy programs to guide parents and children in correctly understanding the disease, encourage parents to adopt positive approaches in assisting their children to overcome psychological stress, actively cooperate with treatment, reinforce positive thinking, reduce disease-related stigma, and rebuild self-confidence.

### 4.2 Most prominent behavioral issues in children with hypospadias: anxiety, learning problems, and conduct disorders

This study indicates that among children with hypospadias, behavioral problems are most notably manifested in three dimensions: anxiety, learning difficulties, and conduct disorders. The incidence rate of anxiety in children with hypospadias is 18.9%, predominantly characterized by “fear of new environments and strangers” and “shyness.” This may be associated with feelings of inferiority and a lack of self-acceptance stemming from experiences of frequently wetting pants or being ridiculed by peers for squatting urination after reaching school age. In this study, we surveyed children aged 3 to 17 years. Given that children aged 3 to 5 years have limited gender awareness, the psychological impact of wetting pants or squatting urination is relatively minor. However, among school-aged children and adolescents, the proportion of positive cases in the anxiety dimension is significantly higher. In terms of learning problems, children with hypospadias often exhibit “learning difficulties,” “inattention,” and “a tendency to start tasks but not complete them.” This may be related to psychological stress induced by disease symptoms, which hinders their ability to concentrate. This is also a major concern for parents of affected children. Regarding conduct disorders, the most frequently reported behaviors include “being rude to adults,” “disobedience or reluctant compliance,” and “pouting or sulking.” This may be attributed to parents’ increased leniency and tolerance due to empathy for their child’s suffering from the disease and undergoing multiple surgeries. As a result, children may develop indulged or spoiled personalities during their growing process. It is suggested that parents can learn the knowledge of the impact of negative psychology on behavioral problems, strengthen the attention and communication with children, understand the psychological status of children, timely guide children to correctly relieve the psychological pressure brought by the disease, and do not indulge children’s inappropriate behavior because of the disease.

### 4.3 Analysis of influencing factors for behavioral problems in children with hypospadias

#### 4.3.1 Number of surgeries

Surgery represents the sole treatment option for congenital hypospadias (3). The recommended optimal timing for surgical intervention is typically between 6 and 18 months after birth (27). However, in clinical practice, some children with hypospadias do not receive their initial evaluation until they exceed the recommended surgical age, with some even undergoing their first hypospadias repair surgery at the age of 17. Some families believe that, as the penis is a reproductive organ, treatment should be deferred until the child has completed physical development. Additionally, left-behind children may experience delayed diagnosis due to the absence of parents who could identify the condition. Postoperative care for hypospadias repair is challenging, with complication rates ranging from 12% to 24%, predominantly occurring within the first year after surgery. Long-term complications can reach as high as 50% (28). Among these, urethral fistula is the most common complication, with an incidence rate of 15% to 30%, often necessitating reoperation (29). The older the age at the time of the first surgery, the higher the incidence of complications and the likelihood of requiring multiple surgeries (30), leading to lower parental satisfaction (31). Surgery, as a negative stimulus, can induce adverse hospital experiences for children. Anxiety, stress, and fear are physiological and psychological responses to perceived threats and are common experiences among most patients undergoing surgery (32). Given that children’s physical and psychological development is not yet mature, they often exhibit behavioral changes when faced with stressful events such as hospitalization and surgery, including trembling, silence, restlessness, or crying. Only a minority can articulate their anxiety and fear verbally (33). Perioperative anxiety and stress can lead to adverse postoperative outcomes, delay recovery, and negatively impact the overall surgical results (34). Furthermore, an increased number of surgeries can exacerbate the impact of postoperative scarring on penile appearance, leading to lower satisfaction levels among children and their families regarding penile aesthetics. This, in turn, may impose additional negative psychological pressure on the children. In this survey, some children had undergone as many as seven urethroplasty procedures.

To address this issue, it is crucial to enhance public awareness and education about hypospadias, particularly in less developed rural areas. This will enable more people to understand the condition and recognize the importance of early and proactive treatment. Efforts should be made to perform surgical corrections within the internationally recommended age range of 6 to 18 months to minimize the psychological impact of the disease on children’s development. Additionally, healthcare professionals can strengthen postoperative education and follow-up care, providing hands-on guidance to parents on home care after surgery and monitoring the children’s postoperative recovery to reduce complications arising from improper care and lower the risk of reoperation.

#### 4.3.2 Urination status

The primary impact of hypospadias on children’s daily lives manifests in their urination status. Due to the abnormal urethral opening, children with hypospadias excrete urine through an atypical orifice, resulting in a lack of control over the direction of the urine stream (35). This often leads to wetting their pants, and in more severe cases, some children may consistently adopt a squatting position to urinate. Upon entering school, these children may experience a blow to their self-esteem due to frequent incidents of wetting their pants and the necessity of squatting to urinate. Consequently, they may develop resistance to urinating and drinking water at school, and in extreme cases, even refuse to attend school altogether. The findings of this study indicate that urination patterns serve as an influencing factor for behavioral problems in these children, whereas the classification of the disease does not appear to be a significant factor. This discrepancy may be attributed to the children’s urination habits and family education. During the investigation, it was observed that the severity of the disease classification did not correlate positively with urination difficulties. In other words, children with more severe disease classifications did not necessarily experience a higher frequency of wetting their pants. This could explain why the disease classification does not have a statistically significant negative impact on the children’s lives. To address this issue, it is essential to guide children and their families in developing a correct understanding of the disease. They should be made aware that having the condition is not their fault. Promoting open communication between children and their families is crucial. Families can provide proper guidance to help children learn how to control the direction of their urine stream, thereby reducing the frequency of wetting their pants. This, in turn, can alleviate the sense of shame associated with the disease, enabling children to urinate and drink water normally at school and actively cooperate with treatment.

## 5 Strengths and limitations

A strength of our study is the fact that all the patients were operated on and followed up at the same hospital, effectively controlling variables related to medical procedures. Furthermore, the consistent research team’s data collection minimized bias from different investigators. Notably, the inclusion of children with hypospadias aged 3–17 years revealed distinct behavioral manifestations across the different age groups. This suggests future research could implement age-specific interventions to enhance treatment precision and effectiveness.

There are several limitations to our study. One of the limitations is the exclusive use of data from children with hypospadias who opted for surgical treatment at hospitals, without establishing a control group of non-treatment participants. It would be difficult to carry out the parents who those unwilling to participate the treatment, meaning the study findings may not reflect the behavioral issues experienced by all children with hypospadias. Children without treatment might continue enduring disease-related impacts, potentially developing more severe behavioral problems. Furthermore, parents who choosing treatment typically demonstrate greater awareness and positivity, which could influence children’s attitudes toward their illness. Additionally, some data were collected through self-reports from parental narratives, which may introduce recall bias. Future research could incorporate more objective measurement tools to improving the data accuracy and reliability.

## 6 Conclusion

The incidence rate of behavioral problems among children with hypospadias is 23.8%, primarily manifesting in three dimensions: anxiety, learning difficulties, and conduct issues. Children who undergo multiple surgeries and frequently wet their pants are more prone to developing behavioral problems. In clinical practice, it is crucial to pay attention to the psychological well-being of these children, as psychological support holds equal importance to clinical expertise.

This study is a single-center cross-sectional survey, and there may be certain biases in the selection of the survey subjects. Therefore, it is recommended that future research adopts high-quality cohort studies to explore the changing patterns of behavioral problems in children with hypospadias over the course of the disease and its treatment. Additionally, further analysis should be conducted to assess the impact of these behavioral problems on the quality of life of both the affected children and their families.

## Data Availability

The original contributions presented in the study are included in the article/Supplementary material, further inquiries can be directed to the corresponding author.
